# An Output-Capacitorless Low-Dropout Regulator with Slew-Rate Enhancement

**DOI:** 10.3390/mi13101594

**Published:** 2022-09-25

**Authors:** Shenglan Ni, Zhizhi Chen, Chenkai Hu, Houpeng Chen, Qian Wang, Xi Li, Sannian Song, Zhitang Song

**Affiliations:** 1 State Key Laboratory of Functional Materials for Informatics, Shanghai Institute of Microsystem and Information Technology, Chinese Academy of Sciences, Shanghai 200050, China; 2 Schools of Microelectronics, University of Chinese Academy of Sciences, Beijing 100049, China

**Keywords:** low-dropout regulator (LDO), output-capacitorless, push–pull, slew-rate enhancement

## Abstract

A novel output-capacitorless low-dropout regulator (OCL-LDO) with an embedded slew-rate-enhancement (SRE) circuit is presented in this paper. The SRE circuit adopts a transient current-boost strategy to improve the slew rate at the gate of the power transistor when a large voltage spike at the output is detected. In addition, a feed-forward transconductance cell is introduced to form a push–pull output structure with the power transistor. The simulation results show that the maximum transient output voltage variation is 23.5 mV when the load current $I_{LOAD}$ is stepped from 0 to 100 mA in 100 ns with a load capacitance of 100 pF, and the settling time is 1.2 μs. The proposed OCL-LDO consumes a quiescent current of 30 μA and has a dropout voltage of 200 mV for the maximum output current of 100 mA.

## 1. Introduction

Power management units are popular in system-on-chip (SoC) applications because multiple voltage regulators can be used to individually power system sub-modules [1]. Among the many candidates for on-chip power management, LDO (low dropout regulator) regulators capable of providing accurate and clean supply voltages are considered suitable for SoC applications. Traditional LDOs rely on large off-chip capacitors on the order of μF at the output to ensure system stability while improving transient response and power supply rejection (PSR) [2,3,4]. For portable systems with SoC architectures, bulky off-chip capacitors are not desirable. This led to the development of LDO regulators without off-chip capacitors at the output [5,6,7].

For portable electronic devices, the low quiescent power consumption of OCL-LDOs is critical for improving power efficiency to extend battery runtime. However, OCL-LDOs trade off power consumption and other performance metrics such as loop stability and dynamic performance [8]. The ability to drive large load currents while achieving low dropout voltage requires a PMOS (positive channel metal oxide semiconductor) transistor with a large size as the power device. Since the gate capacitance of the power transistor is proportional to its width, on the one hand, a low-frequency pole is introduced into the system, which affects the stability of the OCL-LDO, and on the other hand, the time for charging and discharging the gate parasitic capacitance of the power transistor is greatly increased. Especially for applications that require low power consumption, the system faces the problem of reduced bandwidth and slew rate, so improving the transient performance of OCL-LDOs is one of the main design challenges.

Currently, many LDO regulators without large off-chip capacitors have been reported. To cater to the need for the low-power consumption of portable devices in standby mode, some LDOs are designed to operate at currents in the order of nA [9,10]. LDOs with nA bias currents struggle to respond quickly to the load transitions because unity-gain bandwidth (UGB) is limited by ultra-low currents. In addition, low power consumption undoubtedly reduces the slew rate at the gate of the power transistor, further deteriorating the transient response. Reference [11] uses an advanced Q-reduction technique to improve UGB, but the proposed LDO requires a compromise on minimum load current, which limits its application in long-standby systems. Although flipped voltage follower (FVF)-based LDO regulators are easy in transient response, the tradeoff is low loop gain [12,13]. Low loop gain tends to induce poor load regulation [14]. Other LDOs designed with a two-stage amplifier structure also suffer from low gain, especially when operating at low supply voltages [15,16,17,18]. In [19,20,21], adaptive biasing techniques are adopted to improve the transient response of the LDO while maintaining low quiescent power consumption at light loads. However, this solution only works when switching from a heavy load to a light load. Dynamic biasing techniques use capacitive coupling to increase the bias current during load switching, so as to improve the transient performance without increasing steady-state power [9]. Unfortunately, RC networks need to occupy chip areas, and more seriously, the SRE circuit may degrade loop stability.

Since it is difficult for portable applications to balance loop stability and transient response performance at low power consumption, a new solution is required to design OCL-LDOs. This paper proposes a dynamic SRE technique to address the above difficulties. This technique achieves transient enhancement by increasing the slew rate at the gate of the power transistor and the output node during the transient instant. The proposed SRE circuit reuses the frequency compensation capacitors and the common gate transistors, which greatly reduces the additional bias current.

The rest of the paper is organized as follows: Section 2 presents the architecture as well as the stability analysis of the proposed OCL-LDO. Section 3 describes the schematic of the proposed circuit and explains the operation of the circuit during load transitions. Section 4 presents the simulation results, discussions, and performance comparisons. Finally, we draw conclusions in Section 5.

## 2. Proposed Architecture

### 2.1. Topology

**Figure 1:**
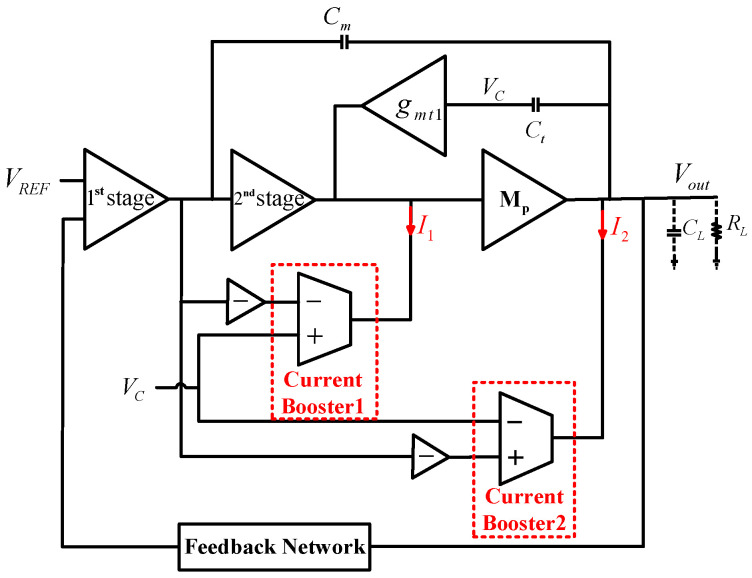
Conceptual structure of the proposed OCL−LDO regulator.

The topology of the proposed OCL-LDO is shown in Figure 1, including an error amplifier as the first stage, a non-inverting amplifier as the second stage, a power transistor as the third stage, a frequency compensation network, a transient-current boosting circuit, and a feedback network, where the compensation network consists of $C_{m}$, $C_{t}$, and $g_{mt1}$, and the transient-current boosting circuit consists of two current boosters. $R_{L}$ represents the effective output resistance. The total capacitance at the output is the equivalent output lumped capacitance of the load capacitor $C_{L}$ in the range of 0–100 pF plus the equivalent parasitic capacitance of the power transistor. The input voltage of the transconductance cell $g_{mt1}$ is denoted as $V_{C}$. In the proposed architecture, the frequency compensation capacitors $C_{m}$ and $C_{t}$ couple the output voltage variation during the load transients and pass it to the current boosters for transient enhancement.

The transient-current boosting circuit consists of two current boosters, as shown in Figure 1. The output current $I_{1,2}$ is quadratically dependent on the booster-cell differential input voltage. Due to the action of the two inverters, the voltages at the positive and negative inputs of the current boosters always change in opposite directions during transients. That is to say, when the voltage at the positive input terminal of booster 1,2 changes by ΔV, the voltage at the negative input terminal changes by −ΔV, then the total input voltage change is $\Delta V_{in1,2}=2\cdot \Delta V$. Therefore, even with small bias currents, $I_{1}$ and $I_{2}$ are able to be boosted up during load transients, which means that the slew rate at the power transistor gate and the output node can be enhanced.

### 2.2. Stability Analysis

**Figure 2:**
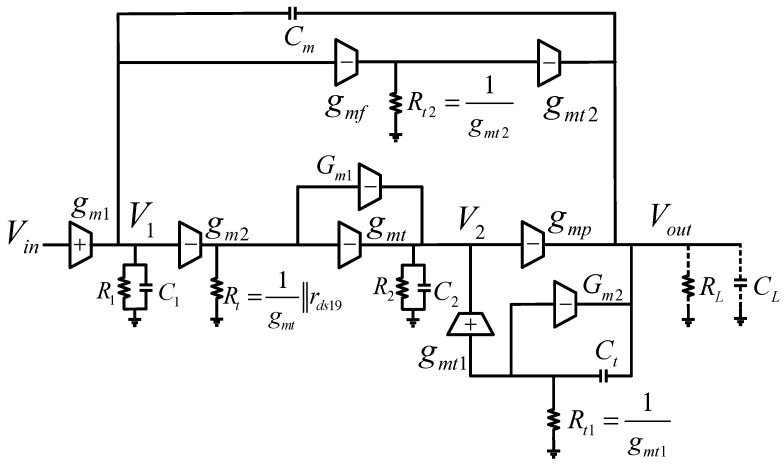
Small−signal model of the proposed OCL−LDO regulator.

The stability of the proposed OCL-LDO is achieved by the TCFC compensation technique, which can provide higher current-bandwidth efficiency [22]. Figure 2 shows the equivalent small-signal model of the proposed OCL-LDO, where $g_{mi}$ is defined as the transconductance of each stage, whereas $R_{i}$ and $C_{i}$ represent the output resistance and lumped parasitic capacitance, respectively. $g_{m2}$ and $g_{mt}$ compose the non-inverting second stage. $r_{ds19}$ is the output resistance of M19, which is a pFET in saturation. $g_{mp}$ is the transconductance of the power transistor Mp. The effective output resistance is defined by $R_{L}=R_{o}∥R_{LOAD}$, where $R_{o}$ and $R_{LOAD}$ is the output resistance of the output stage and load resistance, respectively.$\;C_{L}$ models the load capacitance as defined above. The Miller compensation capacitor $C_{m}$ forms an external feedback loop, and the internal compensation capacitor $C_{t}$ feeds back the output signal to the gate of the power transistor through the transconductance $g_{mt1}$. In order to improve the transient performance of the system, a feed-forward transconductance stage $g_{mf}$ is introduced in the OCL-LDO, which can form a push–pull structure with the power transistor to further improve the slew rate at the output node.

**Figure 3:**
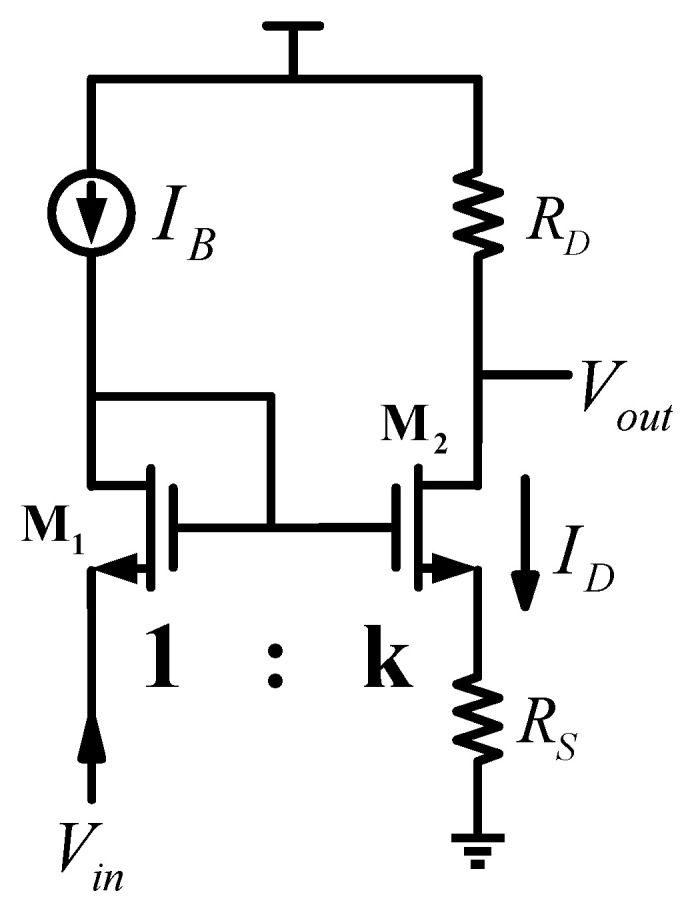
Equivalent model of the transconductance cell $G_{m}$.

Both $G_{m1}$ and $G_{m2}$ are given by the equivalent transconductance $G_{m}$ of the circuit structure shown in Figure 3. $G_{m}$ is defined as:

(1) $$G_{m}=\frac{\partial I_{D}}{\partial V_{in}},$$

**Figure 4:**
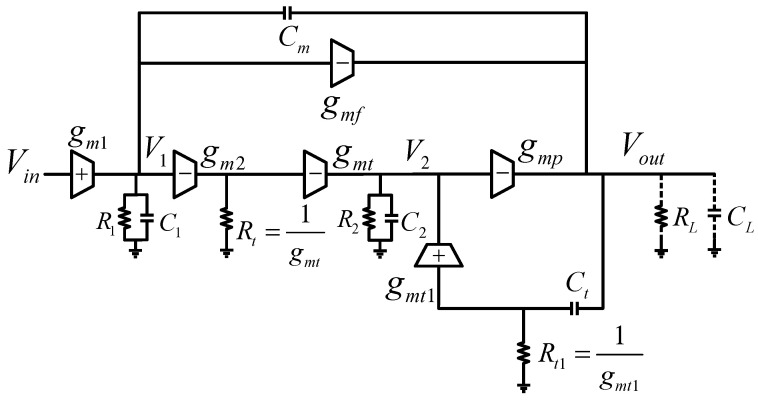
Simplified small−signal model of Figure 2.

$G_{m}$ can be deduced as follows:

(2) $$G_{m}=\frac{g_{m}}{1+g_{m}R_{s}},$$

where $g_{m}$ is the transconductance of M2. In the proposed design, $R_{s}$ is actually realized by the $r_{ds}$ of M15 and M21, which are two nFETs in saturation, showing large resistance, so $g_{m}R_{s}\gg 1$. Specifically, $G_{m1}=\frac{g_{mt1}}{1+g_{mt1}r_{ds15}}$, $G_{m2}=\frac{g_{mt2}}{1+g_{mt2}r_{ds21}}$. It can be concluded that $G_{m1}\approx \frac{1}{r_{ds15}}$, $G_{m2}\approx \frac{1}{r_{ds21}}$. Compared with $g_{mt}$ and $g_{mt1}$, the contributions of $G_{m1}$ and $G_{m2}$ to the current are insignificant and therefore can be ignored. Thus, the small-signal model in Figure 2 can be simplified as shown in Figure 4.

For simplicity, we assume that the DC gain of each stage is large enough, and the compensation capacitance $C_{m}$ is larger than the parasitic capacitance $C_{1}$ of the first stage. $C_{m}$ and $C_{t}$ are much smaller than the load capacitance $C_{L}$, as given by:

(3) $$g_{m1}R_{1},\;g_{m2}R_{2},\;g_{mp}R_{L}\gg 1$$

(4) $$C_{m}\gg C_{1};\;C_{m},C_{t}\ll C_{L}$$

It is worth noting that $C_{2}$ includes the gate parasitic capacitance of the power transistor and is therefore large. The derived small-signal transfer function for the open-loop gain of the OCL-LDO is given by:


(5) $$A_{v}(s)\approx \frac{A_{dc}\left(1+s\frac{g_{m2}g_{mp}C_{t}}{g_{m2}g_{mp}g_{mt1}}-s^{2}\frac{g_{mt1}C_{m}C_{t}}{g_{m2}g_{mp}g_{mt1}}-s^{3}\frac{C_{2}C_{m}C_{t}}{g_{m2}g_{mp}g_{mt1}}\right)}{\left(1+\frac{s}{\left|p_{-3dB}\right|}\right)\left(1+s\frac{g_{m2}g_{mp}C_{t}+g_{mp}g_{mt1}C_{t}}{g_{m2}g_{mp}g_{mt1}}+s^{2}\frac{g_{mt1}C_{2}C_{L}R_{L}+C_{2}C_{t}}{g_{m2}g_{mp}g_{mt1}R_{L}}+s^{3}\frac{C_{2}C_{t}C_{L}}{g_{m2}g_{mp}g_{mt1}}\right)}$$

$A_{dc}$ and $p_{-3dB}$ are the low-frequency gain and the dominant pole, respectively, which are given as:

(6) $$A_{dc}=g_{m1}g_{m2}g_{mp}R_{1}R_{2}R_{L}$$

(7) $$p_{-3dB}=-\frac{1}{g_{m2}g_{mp}R_{1}R_{2}R_{L}C_{m}}.$$

Hence, the gain-bandwidth product (GBW) can be obtained as:

(8) $$\mathrm{GBW}=\frac{g_{m1}}{C_{m}}.$$

Since the load current will change, the stability of the proposed LDO should be discussed for different load conditions.

Case I (low output current): In this case, $R_{L}$ is very large, so that $g_{mt1}C_{2}C_{L}R_{L}\gg C_{2}C_{t}$. The non-dominant poles and zeros can be expressed as:

(9) $$p_{1}=-\frac{g_{mt1}}{\left(g_{m2}+g_{mt1}\right)}\cdot \frac{g_{m2}}{C_{t}},$$

(10) $$p_{2}=-\frac{\left(g_{m2}+g_{mt1}\right)}{g_{mt1}}\cdot \frac{g_{mp}C_{t}}{C_{2}C_{L}},$$

(11) $$p_{3}=-\frac{g_{mt1}}{C_{t}},$$

(12) $$z_{1}=-\frac{g_{mt1}}{C_{t}}$$

(13) $$z_{2}=\frac{g_{m2}g_{mp}}{g_{mt1}C_{m}},$$

(14) $$z_{3}=-\frac{g_{mt1}}{C_{2}}.$$

From the above analysis, it can be seen that $p_{3}\;\mathrm{and}\;z_{1}$ can cancel each other out. The other two zeros, $z_{2}$ and $z_{3}$, only appear at high frequencies. For a third-order Butterworth frequency response with the damping factor $\zeta =\frac{1}{2Q}=0.707$, the stability conditions are given by:

(15) $$p_{2}=2p_{1}=4\mathrm{GBW}$$

When $\frac{g_{m2}}{g_{m1}}$ and $\frac{g_{mp}}{g_{m1}}$ are large, Equation (15) is easily satisfied. It can be noticed that $p_{2}$ is proportional to $g_{mp}$, so the worst stability of the circuit occurs with no load current and maximum load capacitance. As the load current increases, $p_{2}$ will undoubtedly be pushed to higher frequencies and the phase margin will increase.

Case II (moderate to maximum output current): In this case, $R_{L}$ is small, as it is greatly affected by the load current ($R_{L}\propto \frac{1}{I_{LOAD}}$). The expressions for the zeros, dominant pole, and GBW remain the same. The non-dominant poles change, as given by:

(16) $$p_{1}=-\frac{g_{mt1}}{\left(g_{m2}+g_{mt1}\right)}\cdot \frac{g_{m2}}{C_{t}},$$

(17) $$p_{2}=-\frac{\left(g_{m2}+g_{mt1}\right)g_{mp}R_{L}}{C_{2}}$$

(18) $$p_{3}=-\frac{1}{R_{L}C_{L}}$$

It can be observed that $p_{1}$ remains the same. Since GBW does not vary with the load current, $p_{1}=2\;\mathrm{GBW}$ still holds. With a small $R_{L}$, $p_{3}$ is located at a higher frequency than GBW and has no effect on LDO stability. Hence, the loop stability only depends on the location of $p_{2}$. Compared to the case discussed before, even though $R_{L}$ is smaller, the larger $g_{mp}$ pushes $p_{2}$ to higher frequencies, thus improving the phase margin. Furthermore, the zero $z_{1}$ is located slightly beyond the GBW for the enhancement of the phase margin.

In fact, the stability of the circuit is improved with SRE. Specifically, we return to Figure 2 for a detailed analysis of the true equivalent transconductance $g_{m2}\prime$ of the second gain stage. It follows that $g_{m2}\prime =g_{m2}R_{t}\cdot \left(G_{m1}+g_{mt}\right)$, where $R_{t}=\frac{1}{g_{mt}}∥r_{ds19}$. It can be found that $g_{m2}<g_{m2}\prime$, which means that when the SRE circuit fails and the system is under a light load, $p_{1}$ and $p_{2}$ will move closer to the unit gain bandwidth and the stability of the circuit will be slightly worse. At heavy loads, this situation is improved, as $p_{2}$ is still pushed to high frequencies.

## 3. Design of the Proposed OCL-LDO Regulator

### 3.1. Schematic

**Figure 5:**
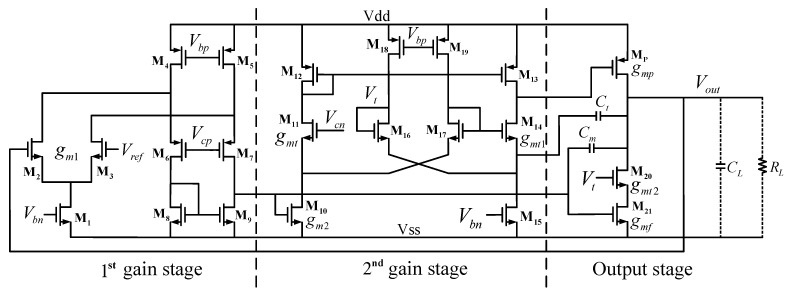
Full schematic of the proposed OCL−LDO regulator.

The full schematic of the proposed OCL-LDO is depicted in Figure 5. The first gain stage is realized by a single folded-cascode error amplifier with M1-M9. The differential pair M2 and M3 provides the transconductance $g_{m1}$. The second stage is a non-inverting amplifier composed by M10–M19. Mp is the power transistor, which together with the feed-forward transconductance module M21 constitutes a push–pull output stage. $C_{m}\;and\;C_{t}$ are capacitors for frequency compensation. $R_{L}$ and $C_{L}$ represent the equivalent output resistance and load capacitance, respectively. The transconductances of transistors M11, M14, M20, and M21 are $g_{mt}$, $g_{mt1}$, $g_{mt2}$, and $g_{mf}$, respectively. $V_{bn}$, $V_{bp}$, $V_{cn}$, and $V_{cp}$ are the bias voltages provided by the bias circuit. The circuit consumes a total of 30 μA quiescent current, of which the first, second, and output stages consume 3 μA, 15 μA, and 9 μA, respectively, and the remaining 3 μA is consumed by the bias circuit.

### 3.2. Overshoot and Undershoot Reduction

The slew rate at the power transistor gate node and output node affects the transient response. As shown in Figure 5, these two nodes correspond to two charging and discharging paths, one is composed of M13 and M14, and the other is composed of $M_{p}$, M20, and M21. Therefore, it is important to dynamically increase the current in these two critical paths. This paper uses the coupling effect of $C_{m}$ and $C_{t}$ when receiving the load current switching request to sense the change of $V_{out}$, and pass it to the two current boosters composed of M14 and M20 to accelerate the charging and discharging of the load capacitor and the gate parasitic capacitance of the power transistor.

When $V_{out}$ generates a spike ΔV in response to an urgent load current request, $C_{m}$ detects the spike and changes the gate voltage of M14 by −ΔV through the inverter formed by M10 and M17, while its source voltage changes ΔV due to the coupling effect of $C_{t}$. This causes the $V_{GS}$ of M14 to change by −2·ΔV. When $V_{out}$ undershoots, the current of M14 is boosted and the current of M13 is decreased through the replication of the current mirror formed by M12 and M13. On the one hand, the second stage can therefore withdraw more current to discharge the gate parasitic capacitance of $M_{p}$. When $V_{out}$ overshoots, the circuit operates in the opposite way to quickly charge the gate capacitance of Mp. On the other hand, for the output node, the push–pull output stage formed by M21 and Mp helps to enhance the slew rate. It should be noted that the path formed by M20 and M21 is the primary channel to discharge the extra current when $V_{out}$ overshoots. Therefore, while reducing the current of Mp, it is more important to increase the current through M20 and M21 to suppress the overshoot of $V_{out}$. Fortunately, M20 can do this by pulling a large current in a similar manner to M14. When $V_{out}$ is regulated back to a steady state, the operation of dynamic current boost is automatically shut down to save energy.

## 4. Simulation Results and Discussions

**Figure 6:**
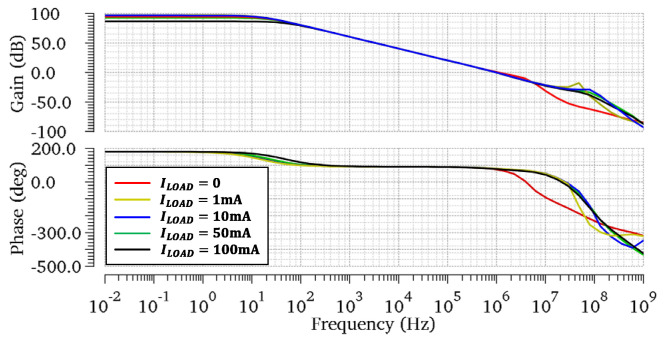
Simulated open−loop gain at different load currents with $C_{L}=100\;\mathrm{pF}$.

**Figure 7:**
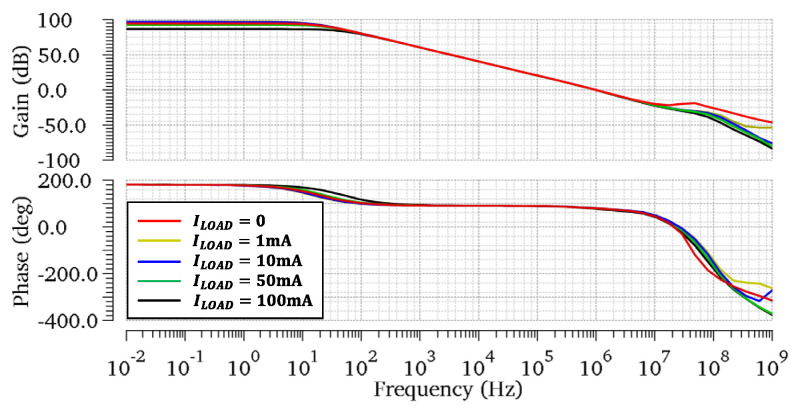
Simulated open−loop gain at different load currents with $C_{L}=0$.

**Figure 8:**
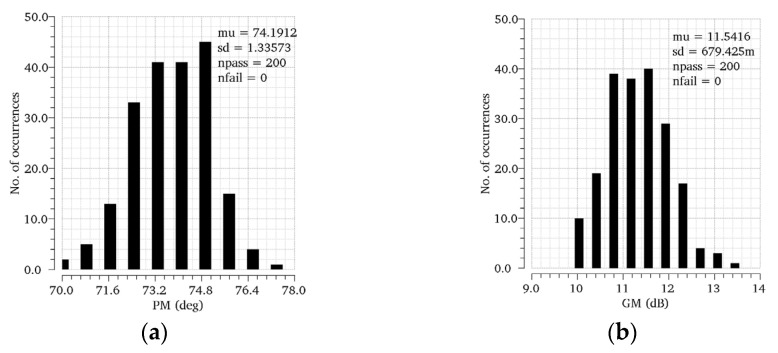
Monte−Carlo simulations when $I_{Load}=0$ and $C_{L}=100\;\mathrm{pF}$. (**a**) PM, (**b**) GM.

The simulated loop gain responses of the proposed regulator at different load current conditions are shown in Figure 6. In the case of $C_{L}=100\;\mathrm{pF}$, the regulator achieves a minimum phase margin (PM) of 74.1° and a minimum gain margin (GM) of 11.2 dB for the load current range from 0 to 100 mA. As the load current raises, the PM and GM increase to 77.2° and 28.1 dB. At heavy load conditions, $R_{L}$ reduces dramatically when Mp enters into the triode region. In this case, the gain of the output stage $g_{mp}R_{L}$ is reduced, as is the $A_{dc}$. However, because the proposed regulator has three gain stages, the minimum $A_{dc}$ of 86.3 dB is found at $I_{LOAD}=100\;\mathrm{mA}$. Moreover, the stability of the proposed OCL-LDO for $C_{L}=0$ is investigated to conduct the loop gain response in Figure 7. A minimum phase margin (PM) of 77.2° and a minimum gain margin (GM) of 21.4 dB are achieved. Theoretical analysis shows that the system has the worst PM and GM when $I_{LOAD}=0$ and $C_{L}=100\;\mathrm{pF}$. Therefore, for further verification, Monte-Carlo simulations are achieved under the condition of $I_{LOAD}=0$ and $C_{L}=100\;\mathrm{pF}$. As Figure 8a,b illustrate, the average PM and GM achieved by the proposed OCL-LDO are 74.2° and 11.5 dB, respectively. Meanwhile, Table 1 shows the simulated PM and GM across PVT variations. The results shown in Figure 8 and Table 1 verify that the stability of the proposed OCL-LDO can be guaranteed.

**Figure 9:**
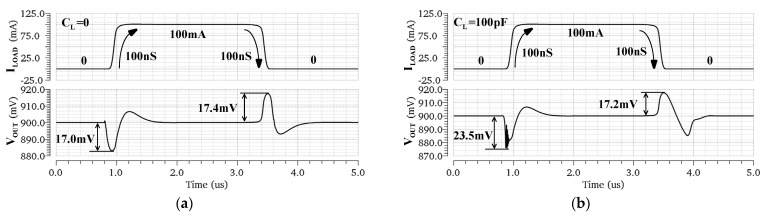
Simulated load transient response of the proposed OCL−LDO regulator for a load current switched between 0 and 100 mA with an edge time of 100 ns. (**a**) $C_{L}=0$, (**b**) $C_{L}=100\;\mathrm{pF}$.

The proposed circuit is able to supply a load current from 0 to 100 mA with a dropout voltage of 200 mV for a supply of 1.1 V. The circuit, including the bias circuit, consumes 30 μA of quiescent current over the specified load current range. The simulated load transient responses under different load capacitor conditions are given in Figure 9. As shown in Figure 9a, when the load current is switched between 0 and 100 mA with an edge time of 100 ns for the case of $C_{L}=0$, the simulated undershoot and overshoot are 17.0 mV and 17.4 mV, respectively. On the other hand, the maximum undershoot and overshoot for $C_{L}=100\;\mathrm{pF}$ are 23.5 mV and 17.2 mV, as shown in Figure 9b. The maximum output voltage variation is about 2.6% (23.5/900 mV) with load step changes of 100 mA/100 ns, and it can return to the final state within 1.2 μs.

Generally speaking, if the output is connected to a large load capacitor, when the load current changes, the overshoot and undershoot can be effectively reduced because the capacitor charges and discharges the output node. However, as shown in Figure 9, the undershoot with 100 pF $C_{L}$ is even larger than the case with 0 pF $C_{L}$. This is because the pole of the output node is close to the unit gain bandwidth when the LDO is connected to a 100 pF load capacitor. During the transition of the load current, the bias voltage and bias current of the amplifier will deviate greatly. In particular, the voltage across the gate and source of M14 deviates sharply due to the change in the opposite direction, resulting in the nonlinear behavior of the circuit. This deviation causes the pole and zero frequency to change during the load transition, so the circuit has more overshoot in this case. On the other hand, the nonlinear behavior of the circuit leads to the generation of rings in the transient response, as shown in Figure 9b. If the gate voltage of M14 is connected to a fixed bias, and the circuit structure, transistor size, and bias current are kept unchanged, the deviation of the bias current of M14 decreases during the load transition. The rings are improved in this case.

**Figure 10:**
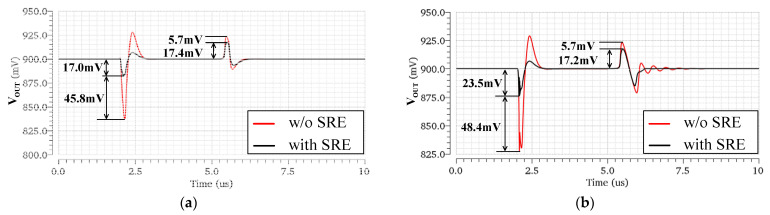
Simulated load transient response of the proposed OCL−LDO regulator under the cases with and without the SRE, for load currents switched between 0 and 100 mA with an edge time of 100 ns. (**a**) $C_{L}=0$, (**b**) $C_{L}=100\;\mathrm{pF}$.

To verify the proposed SRE technique of the OCL-LDO, the transient waveforms of the output voltage are simulated with and without the SRE circuit. For a fair comparison, the only difference is that the gate voltages of the transistors M14 and M20 are biased to a fixed value, while the circuit structure, transistor size, and bias current remain the same. As shown in Figure 10, with the help of the slew-rate-enhancement technique, the undershoot is reduced by more than 45 mV and the settling time is also improved.

It can be seen from Figure 10 that without SRE, the undershoot of the LDO is much larger than the overshoot. This is because when the circuit is switched from light to heavy loads, the gate voltage of the power transistor cannot be pulled down quickly due to the large parasitic capacitance, so it cannot provide a large current to the output in time. To solve this problem, the designed SRE circuit can provide a larger discharge current for the gate capacitance of the power transistor during load transitions. Therefore, the improvement for the undershoot is significantly better compared to the overshoot. Moreover, without SRE, the output has rings when the circuit steps from heavy to light loads, as shown in Figure 10b. This shows that the SRE circuit is helpful to the stability of the system, which is consistent with the previous stability analysis.

**Figure 11:**
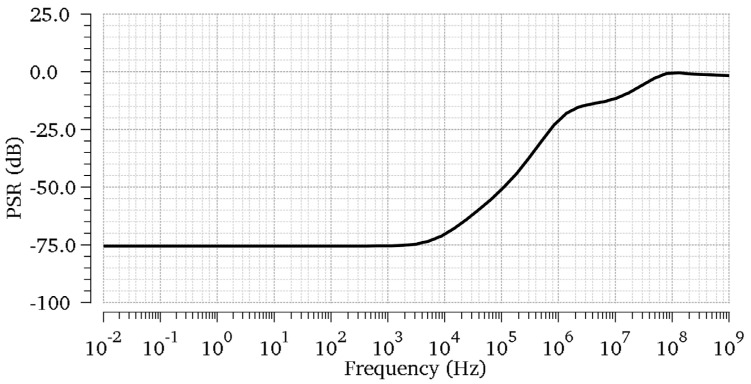
PSR simulations of the proposed OCL−LDO regulator for $I_{Load}=100\;\mathrm{mA}$ and $C_{L}=0$.

Since the PSR is related to the loop gain at low frequencies, and the large load capacitance bypasses the output ripple to the ground at high frequencies, we present the worst-case PSR in Figure 11. As depicted, the PSR has its best value at low frequencies. Because the proposed LDO has a three-stage gain structure and has an optimized gain-bandwidth product in TCFC compensation, the proposed OCL-LDO is capable of providing a good PSR. In order to more objectively evaluate the performance improvement in the proposed OCL-LDO resulting from the slew-rate-enhancement technique, a comparison with the state-of-the-art work is given in Table 2. A figure-of-merit (FOM) for OCL_LDO is adopted to compare the transient performance [23]. Comparisons are also made using a new figure-of-merit ($FOM_{N}$) that takes into account the effects of parasitic capacitances under different processes [14]. It is given by:

(19) $$FOM=K\left(\frac{\Delta V_{out}*I_{Q}}{\Delta I_{LOAD}}\right)$$

(20) $$FOM_{N}=K\left(\frac{\Delta V_{out}*I_{Q}}{\Delta I_{LOAD}*L^{2}}\right)$$

where K is the edge time ratio and defined by:

(21) $$K=\frac{\Delta t\;used\;in\;the\;measurement}{the\;smallest\;\Delta t\;among\;designs\;for\;comparison}.$$

L is the minimum channel length associated with the process. The smaller $FOM_{N}$ value means a better transient performance metric. The $FOM_{N}$ value of the proposed design is second only to that reported in [9]. However, the maximum load capacitance in [9] is only 10 pF, which limits its application. In [17], the dropout voltage of the LDO is designed to be 150mV. Smaller dropout voltage results in higher power efficiency, but at the expense of a larger power transistor for the same drive capability. This means that the gate parasitic capacitance of the power transistor is larger, so the transient response is significantly worse than that of this paper. With the proposed circuit architecture, the voltage-spike detection scheme, and the SRE technique, the transient performance of the designed OCL-LDO has a greater advantage compared to other designs with the same power.

## 5. Conclusions

A low-power OCL-LDO regulator with embedded transient enhancement is implemented with a 40nm standard CMOS process. With the proposed transient enhancement technique and circuit architecture, the OCL-LDO can guarantee stability over the full load range of 0–100 mA without the limitation of a minimum load current. The dropout voltage is 200 mV. The simulation results show that the undershoot of the proposed OCL-LDO is significantly improved, and the quiescent power consumption does not increase when the system is heavily loaded. Compared with the prior art, the proposed OCL-LDO regulator achieves a better transient performance indicator and also provides good performance parameters in terms of line regulation, load regulation, and PSR. The above work will be helpful for on-chip applications.

## Figures and Tables

**Table 1:** Simulation results over PVT variation for the best, mean, and worst stability cases.

	Worst	Mean	Best
PM(deg)	47.8	69.7	77.8
GM(dB)	9.1	16.8	33.1

Including MOS tt/ss/ff/snfp/fnsp corners, R and C tt/ss/ff corners, temperature −40/27/120 ℃, and *V_DD_* 1.1/1.6V.

**Table 2:** Performance comparison with prior-reported OCL-LDO regulators.

Parameters	TCASI [7]	TCASI [9]	TPEL [17]	TPEL [21]	This Work
Year	2007	2018	2020	2022	2022
Technology	0.35 μm	65 nm	65 nm	0.35 μm	40 nm
$V_{DO}\;\left(\mathrm{mV}\right)$	200	200	150	200	200
$V_{out}\;\left(V\right)$	2.8	0.8	0.8	2.5	0.9
$I_{LOAD\left(max\right)}\left(\mathrm{mA}\right)$	50	10	100	100	100
$I_{LOAD\left(min\right)}\;\left(\mathrm{mA}\right)$	0	0	0	0.01	0
$C_{on-chip}\left(\mathrm{pF}\right)$	23	3.9	6	14	8.6
$C_{L}\;\left(\mathrm{pF}\right)$	-	0–10	0–100	0–100	0–100
$I_{Q}\;\left(\mu A\right)$	65	0.1	14	66	30
$\Delta V_{out}\;\left(\mathrm{mV}\right)$	80	231.4	230	255	23.5
$\Delta I_{LOAD}\;\left(\mathrm{mA}\right)$	50	10	100	100	100
Line Reg. (mV/V)	23	N/A	12	0.8	0.2
Load Reg. (μV/mA)	560	1580	90	60	250
PSR (dB)	−57@1kHz	−24@1MHz	−33@10kHz	−41@10kHz	−70@10kHz
Settling Time (μs)	15	0.1	1.2	0.7	1.2
Edge time (ns)	1000	200	220	400	100
Edge time ratio K	10	2	2.2	4	1
FOM (μV)	1040	4.63	70.84	673.20	7.05
$FOM_{N}\;\left(\mu V/\mu m^{2}\right)$	8489.80	1095.86	16766.86	5495.51	4406.25

## Data Availability

Not applicable.
